# Expression of Concern: Curcumin improves age-related and surgically induced osteoarthritis by promoting autophagy in mice

**DOI:** 10.1042/BSR-20171691_EOC

**Published:** 2021-05-14

**Keywords:** autophagy, curcumin, Osteoarthritis

The Editorial Office has been made aware of potential issues surrounding the scientific validity of this paper, hence has issued an expression of concern to notify readers whilst the Editorial Office investigates.

